# Resolving nomenclatural issues pertaining to the Chrysomelinae (Coleoptera, Chrysomelidae) of the Americas and establishing the identity of Demay, 1838

**DOI:** 10.3897/zookeys.1278.175201

**Published:** 2026-04-21

**Authors:** Guillaume J. Dury, Morgan D. Jackson

**Affiliations:** 1 Department of Integrative Biology, University of Texas at Austin, Austin, Texas, USA Department of Integrative Biology, University of Texas at Austin Austin United States of America; 2 Department of Biology, Indiana University Bloomington, Bloomington, Indiana, USA Department of Biology, Indiana University Bloomington Bloomington United States of America; 3 Department of Natural Resources, McGill University, Ste-Anne-de-Bellevue, Quebec, Canada Department of Natural Resources, McGill University Ste-Anne-de-Bellevue Canada

**Keywords:** Authority, International Code of Zoological Nomenclature, leaf beetles, Neotropical region

## Abstract

In reviewing the nomenclature of genus-group names of the Chrysomelinae of the Americas, we uncovered six issues that we resolve here: (1) We explain priority between *Stilodes* and *Leptinotarsa*. Both were described in the same work: Chevrolat, 1836. In synonymizing the two genera, Flowers (2004) became the First Reviser and gave priority to *Stilodes*. We restore genus *Leptinotarsa* Chevrolat, 1836 as distinct from *Stilodes* Chevrolat, 1836. (2) The work in which *Phaedon* Megerle von Mühlfeld, 1823 was described is suppressed for the purposes of zoological nomenclature, making the next person to use the name, Latreille, 1829, the valid authority for *Phaedon*. (3) *Euparocha* Dejean, 1836 is a nomen nudum, and while Motschulsky likely intended to keep Dejean’s spelling, *Euparochia* Motschulsky, 1860 is the correct original spelling. (4) *Lioplacis* Agassiz, 1846 is an unjustified emendation of *Leioplacis* Chevrolat, 1843, and *Lioplacis* Chevrolat is a subsequent usage of the unjustified emendation. *Lioplacis* is not in prevailing usage, therefore *Leioplacis* Chevrolat, 1843 remains the correct original spelling. (5) The accent on Dr Juan Brèthes’ name is often incorrect or missing. Brèthes described *Henicotherus* in volume 32 of the Revista Chilena de Historia Natural. Volume 32 claims it was published in 1928 but was published in 1929. The authority for *Henicotherus* is therefore Brèthes, 1929. (6) The authority previously known only as “Demay”, 1838 refers to Dr Aloysius François De Mey (1793–1870); we provide a short biography, portrait, and updated list of the species described by De Mey, 1838.

## Abstract (es)

Al revisar la nomenclatura de los géneros de Chrysomelinae de las Américas, descubrimos seis problemas que resolvemos aquí: (1) Explicamos la prioridad entre *Stilodes* y *Leptinotarsa*. Ambos fueron descritos en la misma obra: Chevrolat, 1836. Al sinonimizar ambos géneros, Flowers (2004) se convirtió en el Primer Revisor y dio prioridad a *Stilodes*. Restauramos el género *Leptinotarsa* Chevrolat, 1836 como distinto de *Stilodes* Chevrolat, 1836. (2) La obra en la que se describió *Phaedon* Megerle von Mühlfeld, 1823 fue suprimida para los fines de nomenclatura zoológica, por eso la siguiente persona en utilizar el nombre, Latreille, 1829, es la autoridad válida para *Phaedon*. (3) *Euparocha* Dejean, 1836 es un nomen nudum, y aunque Motschulsky probablemente quería mantener la ortografía de Dejean, *Euparochia* Motschulsky, 1860 es la ortografía original correcta. (4) *Lioplacis* Agassiz, 1846 es una enmienda injustificada de *Leioplacis* Chevrolat, 1843. *Lioplacis* no tiene un uso predominante, por lo tanto, *Leioplacis* Chevrolat, 1843 sigue siendo la ortografía original correcta. (5) El acento en el nombre de Brèthes a menudo es incorrecto o falta, el describió *Henicotherus* en el volumen 32 de la Revista Chilena de Historia Natural. El volumen 32 afirma que se publicó en 1928, pero se publicó en 1929. Por lo tanto, la autoridad de *Henicotherus* es Brèthes, 1929. (6) La autoridad anteriormente conocida sólo como “Demay”, 1838 se refiere al Dr Aloysius François De Mey (1793–1870); proporcionamos una breve biografía, un retrato y una lista actualizada de las especies descritas por De Mey, 1838.

## Introduction

We reviewed the spelling, authorities, and years of publication of the genus-group names of the Chrysomelinae of the Americas, and in so doing, we uncovered six nomenclatural issues. We resolve these nomenclatural issues with solutions in line with the rules governing the scientific naming of animals, namely those of the International Code of Zoological Nomenclature (ICZN 1999), hereafter referred to as the Code. We intend for this work to be accessible to even those not familiar with the Code and hope you find the history of these genera and the intricacies of zoological nomenclature approachable and perhaps even enjoyable.

## Why *Stilodes* now has priority over *Leptinotarsa*

The genera *Stilodes* Chevrolat, 1836 and *Leptinotarsa* Chevrolat, 1836 were synonymized by Flowers (2004). Flowers argued that *Stilodes* had priority by virtue of it being an older name because he incorrectly attributed *Stilodes* to Chevrolat, 1843 and incorrectly attributed *Leptinotarsa* to Stål 1858. In reality, *Leptinotarsa* and *Stilodes* are exactly the same age, because for nomenclatural purposes they have the same date of publication: 31 December 1836 (as the exact date of publication is currently unknown, Bousquet and Bouchard 2013).

In Flowers’ defense, *Leptinotarsa* Chevrolat, 1836 was not initially a valid genus but was rather a nomen nudum, Latin for naked name (Bousquet and Bouchard 2013), because it did not meet the criteria of the Code. Specifically, Article 12 states that every new name published before 1931 must be accompanied by a description or a definition of the taxon that it denotes, or by a reference to such a description or definition; names that meet these criteria are considered available. For new genus-group names, being assigned one or more available species names can serve to meet Article 12. The name *Leptinotarsa* Chevrolat, 1836 was considered unavailable (naked) because, when it was first proposed, none of the species names included in it were available.

However, for stability reasons, *Leptinotarsa* Chevrolat, 1836 was made available and conserved by the International Commission of Zoological Nomenclature (hereafter called the Commission) in Opinion 1290 (ICZN 1985). The Commission set aside all type designations made prior to that of Motschulsky, 1860 who assigned *Leptinotarsa heydeni* Stål, 1858 as the type species. Conversely, *Stilodes* Chevrolat, 1836 was valid from its original description. Chevrolat included seven species, but only *Chrysomela humeralis* Gory, 1833 was available; the genus was therefore defined by monotypy as detailed by Bousquet and Bouchard (2013).

Daccordi (2008) and Daccordi and Zoia (2017) noted Flowers’ error and attempted to rectify it. They implied that because *Leptinotarsa*’s description on page 397 appears earlier in Dejean’s second catalogue, *Leptinotarsa* had priority over *Stilodes*, whose description appears later on page 403. Neither relative position in a taxonomic work nor page number are valid reasons for determining priority according to the Code.

Instead, when priority cannot be determined objectively between synonyms—as in the case of the synonymized *Stilodes* Chevrolat, 1836 and *Leptinotarsa* Chevrolat, 1836—Article 24.2.2. of the Code establishes that the First Reviser chooses which synonym has priority (ICZN 1999). No author had placed these genera in synonymy before Flowers (2004); therefore in stating that *Stilodes* had priority by virtue of being older, he, as First Reviser, gave priority to *Stilodes*, even though his reasoning for doing so was incorrect. Under the Code, an incorrect rationale does not invalidate the act of a First Reviser. Since Flowers (2004), most authors have rejected the synonymy and ignored the publication (e.g., Andrews and Gilbert 2005; Löbl and Smetana 2010; Benítez-García et al. 2017; Ordóñez-Reséndiz and López-Pérez 2021; Bezděk and Sekerka 2024); others have cited Flowers (2004) but rejected the synonymy (e.g., Daccordi 2008; Daccordi and Zoia 2017; Flinte et al. 2017). Few have accepted Flowers’ proposal. Chaboo and Flowers (2015) re-iterated the synonymy between the genera but followed Daccordi (2008) in giving priority to *Leptinotarsa*, contradicting Flowers’ previous designation. This nomenclatural act can be ignored; Flowers himself, as First Reviser, established that *Stilodes* has priority.

In the case of subjective synonyms, i.e., synonyms with different type species, the synonymy is open to taxonomic judgement. Molecular phylogenetic data published after 2004 indicate that *Stilodes* Chevrolat, 1836 and *Leptinotarsa* Chevrolat, 1836 are distinct taxa (figure S1 in Montelongo and Gómez-Zurita 2014; figure 1 in Kirsch et al. 2025). In particular, Dury et al. (2026) include a Bayes factor test that provides extremely strong evidence for rejecting the synonymy based on molecular data. We therefore restore genus *Leptinotarsa* Chevrolat, 1836. This means that *Stilodes decemlineata* (Say, 1824) is an invalid combination and, fortunately for the applied entomological literature, the combination *Leptinotarsa decemlineata* (Say, 1824) is also restored. However, we note that if *Stilodes* and *Leptinotarsa* were ever synonymized again—may that day never come—the priority established by Flowers (2004) as First Reviser would stand, the species concerned would be placed in *Stilodes* rather than *Leptinotarsa*, and the Commission would likely need to preserve *Leptinotarsa* for stability reasons all over again!

## The authority for genus *Phaedon* is Latreille, 1829

The authority for genus *Phaedon* is variously given as Megerle von Mühlfeld, 1823 (Balsbaugh 1983; Daccordi and Lavarini 1993; Daccordi 1994; Andrews and Gilbert 2005; Clark and Gilbert 2019; Ordóñez-Reséndiz and López-Pérez 2021), Dahl, 1823 (Limonta and Colombo 2004; Regalin et al. 2006), or Latreille, 1829 (Daccordi 1982; Daccordi in Seeno and Wilcox 1982; Löbl and Smetana 2010; Bousquet et al. 2013; Ge et al. 2015; Bezděk and Sekerka 2024; Bouchard et al. 2024). As correctly pointed out by Balsbaugh (1983), the name *Phaedon* was first proposed in a book edited by Georg Dahl, published in 1823. The genus appears on page 74 as “*Phaedon* v. M.”; the initials “v. M.” refer to Johann Carl Megerle von Mühlfeld, the inaugural curator of entomology for the Imperial Natural History Cabinet in Vienna, Austria. The book, entitled Coleoptera und Lepidoptera. Ein Systematisches Verzeichniss mit beygesetzten Preisen der Vorräthe (“Coleoptera and Lepidoptera. A systematic directory with attached prices of stocks”), was intended as both a catalog or price list and as a checklist. The authority of *Phaedon* would be Megerle von Mühlfeld, 1823 except for a decision by the Commission.

The Commission considered the case of *Enhydrus* Laporte, 1834 (Coleoptera: Gyrinidae), which was widely used but preceded by *Enhydrus* Macleay, 1825 and by *Enhydrus* Dahl, 1823 (in reality, the authority for this taxon was also Megerle von Mühlfeld, 1823). To preserve *Enhydrus* Laporte, 1834, the Commission chose to suppress both *Enhydrus* Macleay, 1825 and Dahl’s entire book for the purposes of zoological nomenclature (Opinion 710, ICZN 1964). This decision made unavailable all the new names Dahl and Megerle von Mühlfeld had proposed in the book. As detailed in the history of the case, T. J. Spilman objected to suppressing the book; this led C. W. Sabrosky to vote against the proposal, explaining that:

*it is dangerous to suppress the work unless all included new genera have been investigated to see whether suppression would cause any difficulty for them. Suppression of the entire work merely to save*Enhydrus*Castelnau may be poor economy. Let us not create problems that we know not of*. (Sabrosky in ICZN 1964)

The authority of genus *Phaedon* is one such problem of which they did not know. In the end, Sabrosky was the only dissenting vote and the proposal passed with 28 affirmative votes (ICZN 1964). As the original description is in a work suppressed for the purposes of zoological nomenclature and considered unavailable, the authority of the genus goes to the next author to meet the requirements for availability within the literature. As reported in Bouchard et al. (2024), the correct authority for *Phaedon* should thus be Latreille, 1829. Note that the type species of *Phaedon*, *Chrysomela armoraciae* Linnaeus, 1758, was designated by Stephens (1835), not Maulik (1926) as sometimes reported (Bouchard et al. 2024).

## *Euparochia* Motschulsky, 1860 is the correct original spelling

Dejean erected the genus *Euparocha* in 1836, but none of the three originally included species were available, which made *Euparocha* a nomen nudum (Bousquet and Bouchard 2013). Chevrolat next used the name *Euparocha* in 1843, but it was once again a nomem nudum because the proposed type species was not available. In 1860, Motschulsky described *Euparochia* but attributed it to Chevrolat (Chevrolat and Dejean both coined names in Dejean’s second catalogue: Dejean 1836). The difference in spelling—*Euparochia* instead of *Euparocha*—appears to have been unintentional. Given the attribution, Motschulsky likely intended to use the same spelling as Dejean. What does the Code say about such cases? Article 32.2. states that: “The original spelling of a name is the ‘correct original spelling’, unless it is demonstrably incorrect as provided in Article 32.5.” Article 32.5.1. states:

*If there is in the original publication itself, without recourse to any external source of information, clear evidence of an inadvertent error, such as a lapsus calami or a copyist’s or printer’s error, it must be corrected. Incorrect transliteration or latinization, or use of an inappropriate connecting vowel, are not to be considered inadvertent errors*.

In other words, the Code states that the author’s intent is only relevant if the intended spelling can be ascertained in the original publication itself (and then only if the error also is not one of transliteration, or Latinization, or use of an inappropriate connecting vowel). In the case of *Euparochia*, without recourse to an external source of information, in this case Dejean (1836), there is no evidence of an inadvertent error. Therefore, the typographical error should not be corrected according to the Code, and *Euparochia* Motschulsky, 1860 is the correct original spelling.

## *Leioplacis* Chevrolat, 1843 is and remains the correct original spelling

In 1836, Dejean created the name *Leioplacis* but included no available species in the entry for the genus, making the name a nomem nudum (Bousquet and Bouchard 2013). The next author to use *Leioplacis* was Chevrolat in 1843, and he correctly established the name by providing a description for the genus and by designating and describing *Leioplacis klugii* Chevrolat, 1843 as the type species. While neither Dejean nor Chevrolat specified the etymology, *Leioplacis* is likely from Greek “*leio*” (λείο) for smooth or polished and “*plakos*” (πλάκος) meaning flat surface or plate.

Agassiz (1846) listed *Lioplacis* as a synonym of *Leioplacis* (for the publication date of Agassiz, 1846 see Bousquet 2016). In zoological nomenclature, intentional changes to the spelling of taxon names are called emendations. Because Agassiz also listed *Leioplacis* Dejean, the change in spelling was likely deliberate and should be considered an emendation. According to the Code, emendations can either be justified, when the original spelling is demonstrably incorrect according to Article 32.5, or unjustified, when the change in spelling violates the rules of the Code. Agassiz (1846) established many unjustified emendations (Bousquet 2016), e.g., *Epirhinus* for *Epirinus* (Daniel 2019) and *Delognatha* for *Dailognatha* (Patrice and Ivan 2008). *Lioplacis* for *Leioplacis* is also an unjustified emendation. As such, according to Article 33.2.3., *Lioplacis* is considered an available name with its own author and date and is considered a junior objective synonym (i.e., based on the same type material) of the name in its original spelling. Thus, *Lioplacis* Agassiz, 1846 is a junior objective synonym of *Leioplacis* Chevrolat, 1843.

In 1865, Stål published the same emendation of *Leioplacis*, spelling it *Lioplacis* and listing himself as the authority, which we interpret as an explicit statement of intention. Because *Lioplacis* Agassiz, 1846 is available and older, despite Stål’s intent to propose the name himself, *Lioplacis* Stål, 1865 is a subsequent usage of *Lioplacis* Agassiz, 1846. We assume that *Lioplacis* Agassiz, 1846 and *Lioplacis* Stål, 1865 refer to the same taxon, but the assumption should be verified. If the names were found to refer to different taxa, *Lioplacis* Stål, 1865 would become a junior homonym in need of replacement.

Afterwards, some authors have claimed that *Lioplacis* Chevrolat is valid and that *Leioplacis* Chevrolat is its synonym (Weise 1916; Strand 1918; Daccordi 1982; Daccordi in Seeno and Wilcox 1982; Daccordi 1994). In all these cases, uses of *Lioplacis* are subsequent usage of the unjustified emendation *Lioplacis* Agassiz, 1846 despite being attributed to Chevrolat.

Concerning unjustified emendations, Article 33.2.3.1. of the Code states: “when an unjustified emendation is in prevailing usage and is attributed to the original author and date it is deemed to be a justified emendation.” The glossary of the Code defines prevailing usage as “that usage of the name which is adopted by at least a substantial majority of the most recent authors concerned with the relevant taxon, irrespective of how long ago their work was published” (ICZN 1999). To determine whether *Lioplacis* is in prevailing usage, we collated its uses and the uses of *Leioplacis* and grouped them by authors concerned with this genus. We include the authorities the authors cited for the genus and year of description when it was specified (Table 1).

**Table 1.** Usage of *Leioplacis* and *Lioplacis* since the first publication of the genus.

Author concerned with the relevant taxon	* Leioplacis *	*Lioplacis*
**Chevrolat (1843)**: *Leioplacis*Chevrolat 1843, valid.	√	–
**Agassiz (1846)**: established the unjustified emendation *Lioplacis* Agassiz, 1846. *Leioplacis* Dejean, [1836] (text states 1834)**^1^** is also listed.	√*	√*
**d’Orbigny (1861)**: *Leioplacis* Dejean, 1836.	√*	–
**Stål (1865)**: *Lioplacis* attributing it to himself; despite this, it should be considered a subsequent usage of *Lioplacis* Agassiz, 1846.	–	–
**Chapuis (1874)**: *Lioplacis* Stål, [1865] (text specifies the work but not the year).	–	–
**Gemminger and Harold (1874)**: these authors used *Lioplacis* Chevrolat and *Leioplacis* Chevrolat, 1843, which they incorrectly claimed was an emendation.	–	√*
**Scudder (1882)**: *Leioplacis* Dejean [1836] (listed as 1834)**^1^** with *Lioplacis* as a synonym.	√*	–
**Weise (1915)**: *Lioplacis* Stål, [1865] (text does not specify a year).	–	–
**Weise (1916)**: uses both *Lioplacis* Chevrolat and *Leioplacis* Chevrolat, 1843. Reports that *Lioplacis* Chevrolat is valid, (incorrectly) that *Leioplacis* Chevrolat, 1843 is a synonym, and (correctly) that *Lioplacis* Stål, 1865 is a synonym.	–	√*
**Strand (1918)**: *Lioplacis* Chevrolat, 1843, with *Leioplacis* Chevrolat, 1843 and *Lioplacis* Stål, 1865 listed as synonyms.	–	√
**Sherborn (1927)**: *Leioplacis* Dejean, [1836] (text states 1835)**^1^** and specified it to be a nomen nudum, *Lioplacis* Agassiz, 1846 is also listed as an emendation of *Leioplacis* Dejean, 1836.	√*	–
**Neave (1939)**: *Leioplacis* Dejean, [1836] (text states 1835)**^1^**, with *Lioplacis* Agassiz, 1846 listed as an emendation.	√*	–
**Bechyné (1948, 1952, 1954)**: *Leioplacis* Chevrolat, 1843.	√	–
**Blackwelder (1957)**: *Leioplacis* Chevrolat, 1843.	√	–
**Buzzi (1976, 1977, 1983)**: *Lioplacis* Stål, [1865] (text states 1863). These works were cited in the context of biological control of *Baccharis halimifolia* L. (Asteraceae) using *Lioplacis elliptica* (Stål, 1860) by authors not otherwise interested in the taxonomy of leaf beetles (e.g.: McFadyen 1987; Day and McAndrew 2002; Cullen et al. 2012; Fernandes et al. 2022).	–	–
**Daccordi (1982; Daccordi in Seeno and Wilcox 1982, 1994; Daccordi in Flinte et al. 2017)**: *Lioplacis* Chevrolat, 1843, an incorrect subsequent spelling, with *Leioplacis* Chevrolat, 1837 as a synonym; given the confusion surrounding publication dates**^1^** and that the works were by Chevrolat and Dejean, this likely refers to *Leioplacis* Dejean, 1836. Personal communication with Daccordi is cited in several more works that use *Lioplacis* rather than *Leioplacis* (e.g.: Jolivet et al. 2005; Jolivet 2008; Jolivet and Verma 2009, 2010).	–	√
**Reid et al. (2009)**: *Lioplacis* Chevrolat; contrary to the above, these authors do not cite personal communication with Daccordi.	–	√*
**Sampaio and da Fonseca (2023) and Sampaio et al. (2024)**: *Leioplacis* Chevrolat, 1843.	√	–

√ indicates explicit usage with both the authority and year. √* indicates usage with the authority, but incorrect or missing year. – indicates that a given spelling was not adopted. **^1^** this work was later established to have been published in 1836, see Bousquet and Bouchard (2013).

Prevailing usage is not well defined and its meaning is under dispute (Dubois 2010; Welter-Schultes 2013), but it seems fairly clear that the unjustified emendation *Lioplacis* is not adopted by at least a substantial majority of the authors concerned with the taxon (Table 1). Accordingly, *Lioplacis* should not be regarded as being in prevailing usage and has not become a justified emendation; *Leioplacis* Chevrolat, 1843 is therefore maintained as the correct original spelling.

## The year and authority for genus *Henicotherus* are Brèthes, 1929

The genus *Henicotherus* was described in volume 32 of the Revista Chilena de Historia Natural and the name is variously attributed to Brethes, 1929 (Daccordi 1982; Daccordi in Seeno and Wilcox 1982), Bréthes, 1929 (Daccordi 1994; Petitpierre and Elgueta 2012), Brèthes, 1929 (Neave 1939; Blackwelder 1957), and Brèthes, 1928 (Jerez and Pizarro-Araya 2020). This raises two questions: what is the correct accentuation of the name, and what is the correct year of publication?

First, the correct spelling is Brèthes (Rossi Belgrano and Rossi Belgrano 2018), not Brethes or Bréthes. Dr Juan Brèthes was Franco-Argentinian: born in France, he spent and worked most of his life in Buenos Aires, Argentina (Rossi Belgrano and Rossi Belgrano 2018). He wrote mostly in Spanish but continued to write in French throughout his career, at times under the first name Jean instead of Juan (e.g., in Brèthes 1924). Despite those switches, he consistently used the grave accent in his surname (i.e., Brèthes). Others may have incorrectly written his name with the acute accent instead (i.e., Bréthes) because it is the only accent used in Spanish.

Second, Brèthes described genus *Henicotherus* in volume 32 of the Revista Chilena de Historia Natural. For taxonomic purposes, works in volume 32 are generally cited as having been published in 1929 (e.g., Sun and Marshall 2003; Fleming et al. 2014; Dulce et al. 2018) but are sometimes cited as having been published in 1928 (e.g., Jerez and Pizarro-Araya 2020). The front matter of volume 32 states it was put to press on 4 April 1928. However, content from the volume itself clearly indicates that it was printed later. In fact, many instances of later dates appear throughout the volume, with the latest date we found being 15 November 1928 on page 315. Unfortunately, the dating of volumes of the Revista Chilena de Historia Natural has been a source of confusion and frustration (Reed 1946; reprinted in Reed 1964), which led Perez-D’Angello (1971) to present a table covering the year of publication for volumes 1 through 55. While we do not have an exact date of publication for volume 32, it was definitely not published until 1929. Since the exact date of publication is unknown, for nomenclatural purposes, it is considered to have been published 31 December 1929 (Perez-D’Angello 1968, 1971). The correct authority for *Henicotherus* is therefore Brèthes, 1929.

## Demay, 1838 was in fact De Mey, 1838

In the 1838 issue of the Revue Zoologique of the Société Cuvierienne, Félix Édouard Guérin-Méneville reports that “doctor Demay” asked the society to print the extract of a notice he presented at the natural history section of the Congrès de Metz reportedly on 12 September 1837. This notice is reported to have contained 13 descriptions of insects collected in French Guiana, of which only 12 were printed (for a list and current taxonomic status, see Table 2).

**Table 2.** Species described by De Mey 1838 and their current status.

Original name (Family: Subfamily)	Current status	References for current status
*Brachinus melanopterus* (Carabidae: Brachininae)	*Pheropsophus melanopterus* (De Mey, 1838), synonym of *P. aequinoctialis* (Linnaeus, 1763)	Chaudoir (1876)
*Brachinus rivierii* (Carabidae: Brachininae)	*Pheropsophus rivierii* (De Mey, 1838)*	Chaudoir (1876) and Arenas-Clavijo et al. (2022)
*Lampyris guyanensis* (Lampyridae: Lampyrinae)	*Photinus guyanensis* (De Mey, 1838)	Zaragoza-Caballero et al. (2023)
*Cyclocephala rufonigra* (Scarabaeidae: Dynastinae)	*Cyclocephala rufonigra* De Mey, 1838, suspected to be a senior synonym of *C. dorsalis* Burmeister, 1847, itself now a junior synonym of *C. dispar* (Herbst, 1790)	Hielkema and Hielkema (2019)
*Doryphora testudo* (Chrysomelidae: Chrysomelinae)	*Platyphora testudo* (De Mey, 1838)	Sampaio and da Fonseca (2023)
*Galeruca subvittata* (Chrysomelidae: Galerucinae)	*Erynephala subvittata* (De Mey, 1838)	Viswajyothi and Clark (2022)
*Cassida metallica* (Chrysomelidae: Cassidinae)	*Stolas metallica* (De Mey, 1838)	Borowiec (1999)
*Cassida chelidonaria* (Chrysomelidae: Cassidinae)	*Stolas chelidonaria* (De Mey, 1838)	Borowiec (1999)
*Erotylus geurinii* (Erotylidae: Erotylinae)	*Erotylus geurinii* De Mey, 1838**	Alvarenga (1994)
*Erotylus debauvei* (Erotylidae: Erotylinae)	*Cypherotylus debauvei* (De Mey, 1838)***	Skelley (2020)
*Erotylus nigrotibialis* (Erotylidae: Erotylinae)	*Oligocorynus nigrotibialis* (De Mey, 1838)	Lopes (2009)
*Erotylus nigripennis* (Erotylidae: Erotylinae)	*Iphiclus nigripennis* (De Mey, 1838)	Skelley (2020)

* Likely in honor of Sister Anne-Marie Rivier (1768–1838, canonized as Saint Marie Rivier in 2022), who, like De Mey, was persecuted for her Catholic faith during the French Revolution; ** In honor of Félix Édouard Guérin-Méneville (1799–1874); *** In honor of Adam de Bauve, who collected the beetles described by De Mey.

In looking for the complete name of “Demay”, one of the authors (GJD) found the proceedings of the fifth session of the Congrès Scientifique de France (Scientific Conference of France) held in Metz in September 1837 (Congrès Scientifique de France 1838). In the meeting minutes of 12 September, the date cited in the Revue Zoologique, there is no mention of insects or any of the Guianas, but in the meeting minutes of 13 September the following text appears (translated from p. 69):

*The section receives the following communications*:

*1 ° From Mr. de Mey, four insects from Cayenne with the figures and descriptions of several others. [The section] instructs Mr. Lasaulce to report on this work*.

In the minutes for 15 September (translated from pp. 84–85):

*The agenda is [sic] a report by Mr. Lasaulce on the new insects offered to the Conference by Mr. de Mey*.

*The insects described in this leaflet offer to science, in addition to the interest which is attached to novelty and discovery, the one which very beautiful species in curious genera may present, by their most common species*.

*These include*Brachinus melanopterus, Brachinus Rivierii, Lampyris Guyanensis, Gelocephala *[sic]* rufonigra, Doryphora testudo, Galeruca subvittata, Cassida metalica *[sic]*, Cassida chelidonaria, Erotilus *[sic]* Geurini *[sic]*, Erotylus Debauvei, *etc., all insects of [French] Guiana, and brought back from that country by Mr. Adam de Bauve*.

*It would be desirable that the description of these new and interesting species might be inserted in the record of the work of the Conference, where little of this kind is doubtless to be found*.

*Mr. de Mey requests that in the event that the commission does not judge it proper to publish his brief, it should be returned to him, with the insects and the figures accompanying it, so that he may publish them immediately*.

**Figure 1.**
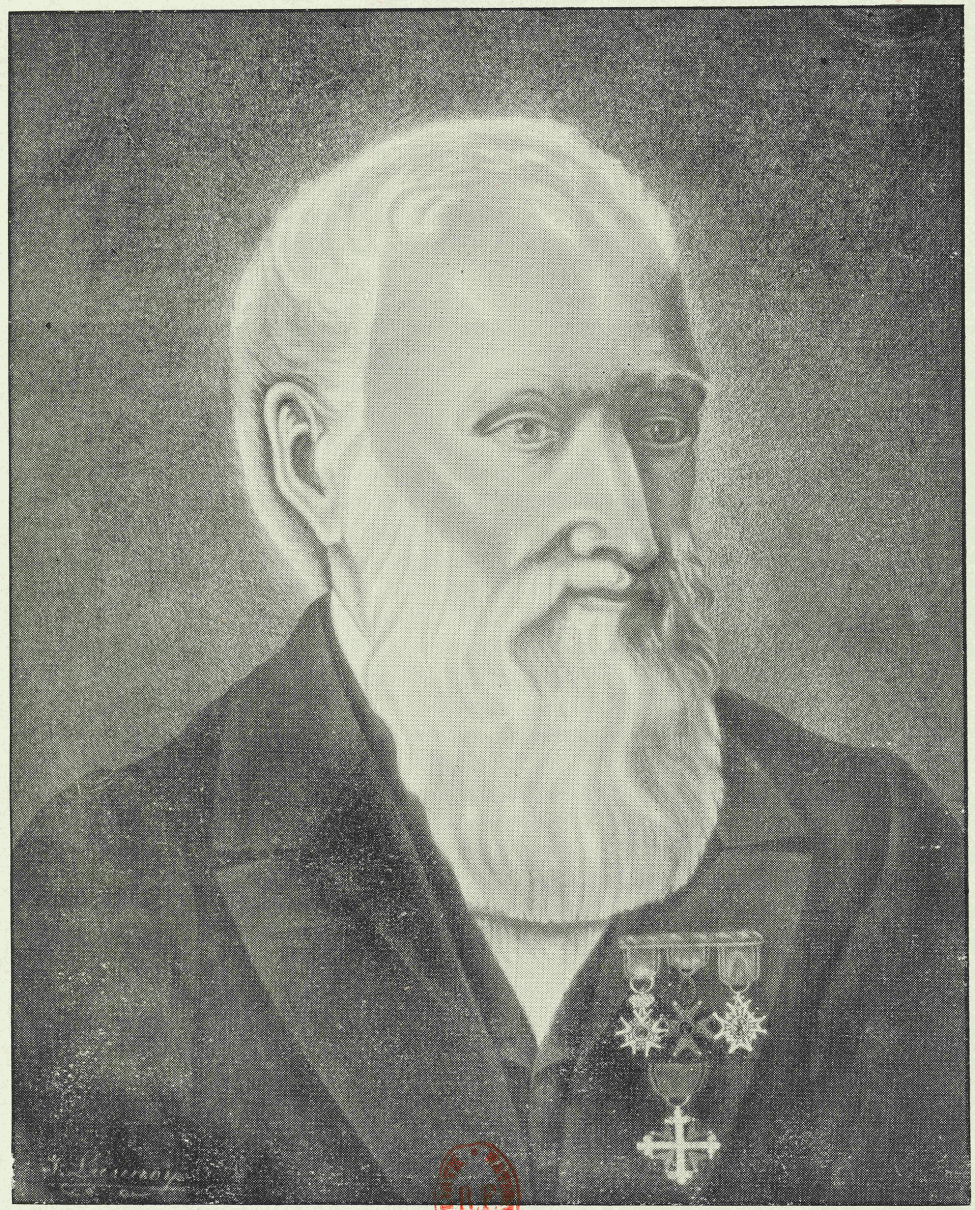
Portrait of Dr Aloysius François De Mey (1793–1870), author cited as “Demay” in Guérin-Méneville (De Mey 1838). De Mey is shown wearing four medals on his lapel: the National Order of the Legion of Honour, the Pontifical Equestrian Order of St. Gregory the Great, the Order of Saint Michael, and the Order of Saints Maurice and Lazarus. The portrait was likely made by Julien François Lannoy (1827–1904), worker at the thermal baths, born in Ypres, a few kilometers from Poperinge, De Mey’s place of birth; both were fervent Catholics and died in Saint-Gervais-les-Bains. The portrait was photographed by Numa Allantaz for Petit (1905), while in the ownership of Mr. Lannoy’s daughter Emélie Marie Lannoy (1880–1955). Its current whereabouts are unknown. (Image in the public domain, credit to the National Library of France, BnF).

While the text does not give a complete name, it does state that the last name is spelled de Mey rather than Demay. Conveniently, the proceedings also included a list of members. The entry on de Mey says: “Mey (de), doctor in medicine, in Paris, member of the geological society of France.” (translated from French). Page 148 of the proceedings includes a first name for him: “Letters on cholera by Mr. Aloysius de Mey” (translated from French). In support of this interpretation, we also found the notice of de Mey becoming a member of the Société Cuvierienne in 1839: “N° 154. Mr. De Mey, medical doctor, member of various learned societies, etc. presented by Mr. Charles d’Orbigny, naturalist helper in geology at the Royal Museum.” (translated from French, Société Cuvierienne 1839). Having both a last name and a first name allowed us to find two biographies for which we provide the translated titles: “Biographical notice on the doctor A.-F. de Mey” by an unknown author (1871) and “The mysticism of the Doctor De Mey: Foundation of his behavior and of his missionary activity at the Baths of St-Gervais” by Dr Eugène Lépinay (1964). The latter biography and the membership notice in the Société Cuvierienne support capitalizing the name as De Mey rather than de Mey. In France, names of French origin and noble names with a single syllable take the particle de, while names of Flemish origin take a capital De (Duneton 2010). Dr De Mey was of Flemish origin, and while he claimed noble origins, his descendants and biographer say he was of common birth (Lépinay 1964). Perhaps it is because Guérin-Méneville correctly doubted De Mey’s nobility that he misspelled the name as Demay; tellingly he also spelled de Bauve as Debauve (we were not able to confirm whether he was indeed noble, but other works spell it de Bauve, e.g., Hurault 1951; Rivière 1966). We provide a portrait (Fig. 1) and a short biographical notice, summarized from the biographies we found (Notice biographique sur le docteur A.-F. de Mey 1871; Lépinay 1964), and historical research we conducted.

### Dr Aloysius François De Mey (1793–1870)

**De Mey, Aloysius François** [sometimes Aloys François, texts published after his death often add a hyphen: Aloysius-François or Aloys-François] (born in Poperinge, West Flanders, Belgium on 25 January 1793 – died on 29 October 1870, in Saint-Gervais-les-Bains, Haute-Savoie, France). Son of Pierre Jacques De Mey and Marie Jeanne Thérèse De Mey (born Uzeel). He received his doctorate of medicine from la Sorbonne in 1821 and was a member of various learned societies including the Société géologique de France, the Société Cuvierienne and the faculté de médecine de Paris. On 23 February 1833, he married Henriette Louise Elizabeth Duchaussoy at the church of Saint-Sulpice in Paris; they never had children. He was a medical doctor, and a supporter of Charles X and the French monarchy. He served as the physician of Henri d’Artois, Duke of Berry, grandson of Charles X, King of France and later, briefly served as royal doctor to Charles X, during the King’s exile in Austria. He was also a devout Catholic and is best known for being the second owner of the thermal baths of Saint-Gervais from 1838 to his death in 1870.

### A note on the provenance and whereabouts of De Mey’s type specimens

Because of confusion regarding where the insects were collected, French Guiana or Guyana, we confirm that all the species described are reported as having been collected in French Guiana by Adam de Bauve (Hielkema and Hielkema 2019 came to the same conclusion). The conference proceedings stated that four of the insects were from Cayenne, French Guiana (without specifying which four) and that all were from French Guiana (Guyane in French); the Revue Zoologique also reports the insects were collected in French Guiana, but Guérin-Méneville adds that the Société Cuvierienne owned almost all the species listed (without specifying which), and that they would enter in a work on the insects collected by de Bauve during a trip to Guyana (translated from French: “during a trip to Demerara and the interior of British Guiana”, both now part of Guyana). It should be noted that Adam de Bauve made several trips to the area; in addition to French Guiana and Guyana, his trips also brought him to Suriname (then Dutch Guiana) and northern Brazil (Abonnenc et al. 1957; Lescure 1989). We are not aware of the work mentioned by Guérin-Méneville being published; therefore, which of the species occur in French Guiana, Guyana, Suriname, and elsewhere remains to be elucidated.

As far as we are aware, the current whereabouts of De Mey’s types are unknown. Unfortunately, in addition to killing approximately 175 people, the outburst flood from the Tête Rousse Glacier—which released an estimated 200,000 m^3^ of water and ice on the night of 11–12 July 1892—also destroyed much of the thermal baths complex, including De Mey’s personal library, his collection of medals, and his natural history collections (Lépinay 1964; Vincent et al. 2010). We can only hope that the type specimens had been removed prior to the catastrophe and may yet be rediscovered.

## Additional information

### Artificial Intelligence (AI) use

The authors accept full responsibility for the content of the manuscript, including the disclosure of any use of AI.

No AI tools were used in the preparation of this manuscript.

### Author ORCIDs

G.J. Dury https://orcid.org/0000-0002-2758-8073

M.D. Jackson https://orcid.org/0000-0001-8574-3097

### Data availability

All of the data that support the findings of this study are available in the main text.

## References

[B1] Abonnenc E, Hurault J, Saban R (1957) Bibliographie de la Guyane Française; Tome 1 ouvrages et articles de langue française concernant la Guyane et les territoires avoisinants. Éditions Larose, Paris, France, 278 pp. http://catalogue.bnf.fr/ark:/12148/cb32595286p

[B2] Agassiz L (1846) Nomenclatoris zoologici. Index universalis, continens nomina systematica classium, ordinum, familiarum et generum animalium omnium, tam viventium quam fossilium, secundum ordinem alphabeticum unicum disposita, adjectis homonymiis plantarum, nec non variis adnotationibus et emendationibus. Jent & Gassmann, Solothurn: Switzerland, 393 pp.

[B3] Alvarenga M (1994) Catálogo dos Erotylidae (Coleoptera) Neotropicais. Revista Brasileira de Zoologia 11: 1–175. 10.1590/S0101-81751994000100001

[B4] Andrews FG, Gilbert AJ (2005) A preliminary annotated checklist and evaluation of the diversity of the Chrysomelidae (Coleoptera) of the Baja California peninsula, Mexico. Insecta Mundi 19: 89–116.

[B5] Arenas-Clavijo A, Montoya-Lerma J, Moret P (2022) Diversity of Geadephaga (Coleoptera: Carabidae and Cicindelidae) in Colombia: an approach from existing literature. Biota colombiana 23: 1–56. 10.21068/2539200X.962

[B6] Balsbaugh EUJ (1983) A taxonomic revision of the genus *Phaedon* North of Mexico (Coleoptera: Chrysomelidae). North Dakota Insects Schafer-Post Series No. 15: 1–71.

[B7] Bechyné J (1948) Notes sur les chrysomélides de l’Amérique du Sud (Col.). Revista de Entomologia 19: 295–312.

[B8] Bechyné J (1952) Nachtäge zu den Katalogen von Blackwelder und Junk-Schenkling der neotropischen echten Chrysomeliden. (Col. Phytophaga). Entomologische Arbeiten aus dem Museum G Frey 1: 1–62.

[B9] Bechyné J (1954) Beitrage zur Kenntnis der echten Chrysomeliden (Col. Phytophaga). Entomologische Arbeiten aus dem Museum G Frey 5: 581–674. 10.5281/zenodo.16480438

[B10] Benítez-García B, López-Pérez S, Zaragoza-Caballero S (2017) Sinopsis de los géneros mexicanos de Chrysomelinae (Coleoptera: Chrysomelidae). Revista Mexicana de Biodiversidad 88: 335–348. 10.1016/j.rmb.2017.03.026

[B11] Bezděk J, Sekerka L (2024) Chrysomeloidea II (Orsodacnidae, Megalopodidae, Chrysomelidae) – Part 1: Updated and Revised Second Edition. Vol. 6/2/1, Brill, Leiden, The Netherlands, 750 pp. 10.1163/9789004443303

[B12] Blackwelder R (1957) Checklist of the coleopterous insects of Mexico, Central America, the West Indies, and South America. Part 3. Bulletin of the United States National Museum 185: 1–1492. 10.5479/si.03629236.185.3

[B13] Borowiec L (1999) A world catalogue of the Cassidinae. Biologica Silesiae, Wrocław, Poland, 476 pp.

[B14] Bouchard P, Bousquet Y, Davies AE, Cai C (2024) On the nomenclatural status of type genera in Coleoptera (Insecta). ZooKeys 1194: 1–981. 10.3897/zookeys.1194.106440 PMC10955229 38523865

[B15] Bousquet Y (2016) Litteratura Coleopterologica (1758–1900): a guide to selected books related to the taxonomy of Coleoptera with publication dates and notes. ZooKeys 583: 1–776. 10.3897/zookeys.583.7084

[B16] Bousquet Y, Bouchard P (2013) The genera in the second catalogue (1833–1836) of Dejean’s Coleoptera collection. ZooKeys 282: 1–219. 10.3897/zookeys.282.4401 PMC3677338 23794836

[B17] Bousquet Y, Bouchard P, Davies A, Sikes D (2013) Checklist of beetles (Coleoptera) of Canada and Alaska. Second edition. ZooKeys 360: 1–44. 10.3897/zookeys.360.4742 PMC3867111 24363590

[B18] Brèthes J (1924) Description d’une Galle de “*Calliandra bicolor*” et de l’Hyménoptère qui la Produit. Revista de la Faculdad de Agronomia de La Plata 15: 23–26.

[B19] Brèthes J (1929) Contribution sur la connaissance des Chrysomélides du Chili. Revista Chilena de Historia Natural 32: 204–220.

[B20] Buzzi ZJ (1976) Nota sobre a biologia e ontogenia de *Lioplacis elliptica* (Stål, 1860) (Coleoptera: Chrysomelidae). Dusenia 9: 139–143.

[B21] Buzzi ZJ (1977) Uma nova especie de *Lioplacis* do sul do Brazil (Coleoptera: Chrysomelidae). Dusenia 10: 229–232.

[B22] Buzzi ZJ (1983) Estagios imaturos e ciclo evolutivo de *Lioplacis caratubae* Buzzi, 1977 (Coleoptera: Chrysomelidae: Chrysomelinae). Revista Brasileira de Entomologia 27: 285–295.

[B23] Chaboo CS, Flowers RW (2015) Beetles (Coleoptera) of Peru: A Survey of the Families. Chrysomelidae: Chrysomelinae. Journal of the Kansas Entomological Society 88: 380–383. 10.2317/kent-88-03-380-383.1

[B24] Chapuis F (1874) Histoire naturelle des insectes. Genera des coléoptères, ou exposé méthodique et critique de tous les genres proposés jusqu’ici dans cet ordre d’insectes. Tome Dixième Famille des Phytophages. Libraire Encyclopédique de Roret, Paris, France, 455 pp.

[B25] Chaudoir M (1876) Monographie des brachynides. Annales de la Société Entomologique de Belgique 19: 11–104.

[B26] Chevrolat LAA (1843) Chrysomélines. In: D’Orbigny CVD (Ed) Dictionnaire universel d’histoire naturelle résumant et complétant tous les faits présentés par les encyclopédies, les anciens dictionnaires scientifiques, les œuvres complètes de Buffon, et les meilleurs traités spéciaux sur les diverses branches des sciences naturelles; donnant la description des êtres et des divers phénomènes de la nature, l’étymologie et la définition des noms scientifiques, les principales applications des corps organiques et inorganiques, à l’agriculture, à la médecine, aux arts industriels, etc; dirigé par M Charles d’Orbigny, et enrichi d’un magnifique atlas de 288 planches gravées sur acier Tome troisième. Renard, Martinet et C., Paris, France, 742 pp. 10.5962/bhl.title.156428

[B27] Clark SM, Gilbert AJ (2019) A New Species of *Phaedon* Megerle von Mühlfeld, 1823 (Coleoptera: Chrysomelidae: Chrysomelinae) from Utah and Colorado, USA. The Coleopterists Bulletin 73: 193–199. 10.1649/0010-065X-73.1.193

[B28] Congrès Scientifique de France (1838) Cinquième session, tenue à Metz, en Septembre 1837. In: Congrès Scientifique de France. Lamort, Metz, France, 634 pp.

[B29] Cullen J, Julien M, McFadyen R (2012) Biological control of weeds in Australia. CSIRO Publishing, 648 pp.

[B30] d’Orbigny CVD (1861) Dictionnaire universel d’histoire naturelle. Tome Cinquième. L. Houssiaux et Cie., Paris, France, 768 pp. 10.5962/bhl.title.156428

[B31] Daccordi M (1982) Chrysomelinae. In: Seeno TN, Wilcox JA (Eds) Leaf beetle genera (Coleoptera: Chrysomelidae). Entomography Publications, Sacramento, CA, USA, 75–95.

[B32] Daccordi M (1994) Notes for the phylogenetic study of Chrysomelinae, with descriptions of new taxa and a list of all the known genera (Coleoptera: Chrysomelidae, Chrysomelinae). In: Furth DG (Ed.) Proceedings of the Third International Symposium on the Chrysomelidae, Beijing, China. Backhuys Publishers, Leiden, The Netherlands, 60–84.

[B33] Daccordi M (2008) The species of *Elytromena* Motschulsky, 1860 with observations on *Elytrosphaera* Chevrolat, 1836 and related genera. In: Memoirs on Biodiversity. World Biodiversity Association Onlus Verona, Verona, Italy, 417–463.

[B34] Daccordi M, Lavarini N (1993) Le specie Italiane del genere *Phaedon* (Coleoptera: Chrysomelidae). Bollettino del Museo civico di Storia Naturale di Verona 17: 481–512.

[B35] Daccordi M, Zoia S (2017) Nuove specie di *Leptinotarsa* Chevrolat, 1836. Bollettino Museo Regionale di Scienze Naturali Torino 33: 251–268.

[B36] Dahl G (1823) Coleoptera und Lepidoptera. Ein Systematisches Verzeichniss mit beygesetzten Preisen der Vorräthe. Gedruckt bey J. E. Akkermann, Vienna, Austria, 105 pp. 10.5962/bhl.title.124180

[B37] Daniel GM (2019) The nomenclatural status of the generic nomina *Epirinus* Dejean, 1833 and *Epirhinus* Agassiz, 1846 (Coleoptera, Scarabaeidae, Scarabaeinae). Bionomina 15: 63–65. 10.11646/bionomina.15.1.6

[B38] Day MD, McAndrew TD (2002) Status of *Charidotis pygmaea* (Coleoptera: Chrysomelidae) as a biological control agent of *Lantana montevidensis* (Verbenaceae) in Australia. Biological Control 23: 27–34. 10.1006/bcon.2001.0983

[B39] De Mey [in Guérin-Méneville FÉ] (1838) II. Travaux inédits. Revue Zoologique 1: 19–24.

[B40] Dejean PFMA (1836) Catalogue des coléoptères de la collection de M. le comte Dejean. Livraison 5. Mequignon-Marvis père et fils, Paris, 443 pp. 10.5962/bhl.title.8771

[B41] Dubois A (2010) Zoological nomenclature in the century of extinctions: priority vs. ‘usage’. Organisms Diversity & Evolution 10: 259–274. 10.1007/s13127-010-0021-3

[B42] Dulce HZ, Jesús R-N, Atilano C-R, José LC-S (2018) Checklist of Tachinidae (Insecta, Diptera) in Mexico. Transactions of the American Entomological Society 144: 1–89. 10.3157/061.144.0113

[B43] Duneton C (2010) L’homme du 18 juin, c’est De Gaulle ou de Gaulle ? Le Figaro 20489: 8.

[B44] Dury GJ, Windsor DM, Sharanowski BJ, Sekerka L, Bede JC (2026) Molecular phylogenetics of Neotropical chrysomeline beetles: Evidence for a constrained history of host plant use. bioRxiv. 10.64898/2026.01.30.702876

[B45] Fernandes GW, Oki Y, Barbosa M (2022) *Baccharis*: from evolutionary and ecological aspects to social uses and medicinal applications. Springer Nature, 578 pp. 10.1007/978-3-030-83511-8

[B46] Fleming AJ, Wood DM, Smith MA, Hallwachs W, Janzen DH (2014) Revision of the New World species of *Houghia* Coquillett (Diptera, Tachinidae) reared from caterpillars in Area de Conservación Guanacaste, Costa Rica. Zootaxa 3858: 1–90. 10.11646/zootaxa.3858.1.1 25283171

[B47] Flinte V, Abejanella A, Daccordi M, Monteiro RF, Macedo MV (2017) Chrysomelinae species (Coleoptera, Chrysomelidae) and new biological data from Rio de Janeiro, Brazil. ZooKeys 720: 5–22. 10.3897/zookeys.720.13963 PMC5784215 29391849

[B48] Flowers RW (2004) The genera of Chrysomelinae (Coleoptera: Chrysomelidae) in Costa Rica. Revista de Biologia Tropical 52: 77–83. 10.15517/rbt.v52i1.14754 17357402

[B49] Ge S-Q, Daccordi M, Ren J, Yang X-K (2015) Revision of *Phaedon* Latreille from China (Coleoptera: Chrysomelidae). Zoological Systematics [动物分类学报] 40: 1–30. 10.11865/zs.20150101

[B50] Gemminger M, Harold E (1874) Catalogus Coleopterorum hucusque descriptorum synonymicus et systematicus. Vol. Tom. XI. Chrysomelidae (Pars I.), A Franck and Williams & Norgate, Paris, France and London, United Kingdom, 242 pp. 10.5962/bhl.title.9089

[B51] Gory HL (1833) Plate 49. In: Guérin-Méneville FÉ (Ed) Iconographie du règne animal de G Cuvier, ou réprésentation d’après nature de l’une des espèces les plus remarquables et souvent non figurées de chaque genre d’animaux Avec un texte descriptif mis au courant de la science Ouvrage pouvant servir d’atlas a tous les traités de zoologie Insectes. J. B. Baillière, Paris, France. 10.5962/bhl.title.10331

[B52] Hielkema AJ, Hielkema MA (2019) An annotated checklist of the Scarabaeoidea (Insecta: Coleoptera) of the Guianas. Insecta Mundi 0732: 1–306.

[B53] Hurault J (1951) A propos des sources de l’Oyapock. Journal de la société des américanistes 40: 254–255.

[B54] ICZN (1964) Opinion 710 *Enhydrus* Laporte, 1834 (Insecta, Coleoptera): Validated under the plenary powers. Bulletin of Zoological Nomenclature 21: 242–245. 10.5281/zenodo.16175070

[B55] ICZN (1985) Opinion 1290 *Leptinotarsa*, Chevrolat, 1837 (Insecta, Coleoptera): Conserved. Bulletin of Zoological Nomenclature 42: 21–23.

[B56] ICZN (1999) International Code of Zoological Nomenclature. The International Trust for Zoological Nomenclature, London, United Kingdom, 306 pp. 10.5962/bhl.title.50608

[B57] Jerez V, Pizarro-Araya J (2020) Una revisión de *Henicotherus* Brèthes, 1928 (Coleoptera, Chryso- melidae, Chrysomelinae); género endémico y áptero de Chile. Gayana (Concepción) 84: 101–111. 10.4067/S0717-65382020000200101

[B58] Jolivet P (2008) La faune entomologique en Nouvelle-Calédonie. Le Coléoptériste 11: 35–47.

[B59] Jolivet P, Verma KK (2009) Biogeography and Biology of the New Caledonian Chrysomelidae (Coleoptera) In: Research on Chrysomelidae, Volume 2. Brill, Leiden, Netherlands, 211–224. 10.1163/ej.9789004169470.1-299.83

[B60] Jolivet P, Verma KK (2010) Good morning Gondwana. Annales de la Société entomologique de France 46: 53–61. 10.1080/00379271.2010.10697638

[B61] Jolivet P, Verma KK, Mille C, d’Entomologie BP (2005) New Observations on the Biology of Chrysomelidae of New Caledonia and Description of Two New Species from the Main Island. Revue française d’Entomologie (NS) 27: 63–72.

[B62] Kirsch R, Okamura Y, García-Lozano M, Weiss B, Keller J, Vogel H, Fukumori K, Fukatsu T, Konstantinov AS, Montagna M, Moseyko AG, Riley EG, Slipinski A, Vencl FV, Windsor DM, Salem H, Kaltenpoth M, Pauchet Y (2025) Symbiosis and horizontal gene transfer promote herbivory in the megadiverse leaf beetles. Current Biology 35: 640–654.e647. 10.1016/j.cub.2024.12.028 39826554

[B63] Laporte FLNC (1834) Etudes entomologiques, ou description d’insectes nouveaux: et observations sur leur synonymie. Première partie. Méquignon-Marvis Père et Fils, Paris, France, 479 pp.

[B64] Latreille PA (1829) Les crustacés, les arachnides et les insectes: distribués en familles naturelles. Vol. Tome Second, Chez Déterville et chez Crochard, Paris, France, 556 pp. 10.5962/bhl.title.11575

[B65] Lépinay E (1964) Le mysticisme du Docteur De Mey: Fondement de son comportement et des ses Actions Missionaires aux Bains de St-Gervais de 1840 à 1870. Mémoires et documents publiés par l’Académie du Faucigny Tome XIV: 37–53. https://gallica.bnf.fr/ark:/12148/bpt6k9651390d

[B66] Lescure J (1989) Des Voyageurs-Naturalistes du Muséum en Guyane II: Lacordaire, Le Prieur et de Bauve; Le Prix d’Encouragement de la Société de Géographie (1830–1839). Nature Guyanaise 2: 14–21.

[B67] Limonta L, Colombo M (2004) Ritrovamento di *Phaedon brassicae* Baly (ColeopteraChrysomelidaeChrysomelinae) in Italia. Bollettino Di Zoologia Agraria E Bachicoltura 36: 369–371.

[B68] Linnaeus C (1758) Systema Naturae per regna tria naturae, secundum classes, ordines, genera, species, cum characteribus, differentiis, synonymis, locis. Vol. Tomus I, Impensis direct, Stockholm, Sweden, 824 pp. 10.5962/bhl.title.542

[B69] Linnaeus C (1763) Centuria Insectorum Rariorum. Uppsala, Sweden, 32 pp. 10.5962/bhl.title.10429

[B70] Löbl I, Smetana A (2010) Chrysomeloidea. Vol. 6, Apollo Books, Stenstrup, Denmark, 924 pp. 10.1163/9789004260917

[B71] Lopes PL (2009) Taxonomia e análise cladística de *Oligocorynus* Chevrolat, 1836 *sensu* Alvarenga (1994) (Coleoptera, Erotylidae, Erotylinae, Erotylini). Ph.D. thesis, São Paulo, Brazil: University of São Paulo, 300 pp. 10.11606/t.41.2009.tde-08072009-093526

[B72] Macleay WS (1825) Annulosa Javanica, or an attempt to illustrate the natural affinities and analogies of the insects collected in Java by Thomas Horsfield, M. D. F. L. & G. S. and deposited by him in the museum of the honourable East-India Company. Vol. 1, Kingsbury, Parbury & Allen, London, United Kingdom, 50 pp. 10.5962/bhl.title.65151

[B73] Maulik S (1926) The Fauna of British India including Ceylon and Burma. Coleoptera, Chrysomelidae (Chrysomelinae and Halticinae). Taylor and Francis, London, United Kingdom, 442 pp. 10.25662/gc09-tz61

[B74] McFadyen PJ (1987) Host specificity and biology of *Lioplacis elliptica* [Col.: Chrysomelidae] introduced into Australia for the biological control of *Baccharis halimifolia* [Compositae]. Entomophaga 32: 19–21. 10.1007/BF02390927

[B75] Montelongo T, Gómez-Zurita J (2014) Multilocus molecular systematics and evolution in time and space of *Calligrapha* (Coleoptera: Chrysomelidae, Chrysomelinae). Zoologica Scripta 43: 605–628. 10.1111/zsc.12073

[B76] Neave SA (1939) Nomenclator Zoologicus: A list of the names of genera and subgenera in zoology from the tenth edition of Linnaeus 1758 to the end of 1935. Vol. 2 (D–L), Zoological Society of London, London, U.K., 1025 pp.

[B77] Notice biographique sur le docteur A.-F. de Mey (1871) Impr. F. Cassagnes, Saint-Julien, France, 49 pp. https://gallica.bnf.fr/ark:/12148/bpt6k6443589p

[B78] Ordóñez-Reséndiz MM, López-Pérez S (2021) Mexican leaf beetles (Coleoptera: Megalopodidae, Orsodacnidae, and Chrysomelidae): new records and checklist. Revista Mexicana de Biodiversidad 92: 1–113. 10.22201/ib.20078706e.2021.92.3873

[B79] Patrice B, Ivan L (2008) Case 3401 *Delognatha* Lacordaire, 1859 (Insecta, Coleoptera): proposed conservation. Bulletin of Zoological Nomenclature 65: 194–197. 10.21805/bzn.v65i3.a16

[B80] Perez-D’Angello V (1968) Bibliografía entomológica chilena. Noticiario Mensual del Museo Nacional de Historia Natural, Chile 139: 3–10.

[B81] Perez-D’Angello V (1971) Análisis de las fechas de aparición de la “Revista Chilena de Historia Natural”. Boletín del Museo Nacional de Historia Natural, Chile 29: 117–120. 10.54830/bmnhn.v29.1971.599

[B82] Petit C (1905) Saint-Gervais de 1805 à 1905. Association typographique, Lyon, France, 54 pp.

[B83] Petitpierre E, Elgueta M (2012) A chromosomal analysis of four species of Chilean Chrysomelinae (Coleoptera, Chrysomelidae). Comparative Cytogenetics 6: 335–340. 10.3897/CompCytogen.v6i4.3519 PMC3834574 24260673

[B84] Reed EP (1946) La Revista Chilena de Historia Natural. Ciencia e Investigación 2: 390–391.

[B85] Reed EP (1964) La Revista Chilena de Historia Natural. Noticiario Mensual del Museo Nacional de Historia Natural, Chile 101: 1–3.

[B86] Regalin R, Bezdek J, Penati FE, Ciapponi L (2006) Catalogo topografico commentato dei Crisomelidi (Insecta, Coleoptera, Chrysomelidae) della provincia di Sondrio (Lombardia, Italia settentrionale). Il Naturalista Valtellinese (Atti del Museo Civico di Storia Naturale di Morbegno) 17: 11–131.

[B87] Reid CAM, Beatson M, Hasenpusch J (2009) The morphology and biology of *Pterodunga mirabile* Daccordi, an unusual subsocial Chrysomeline (Coleoptera: Chrysomelidae). Journal of Natural History 43: 373–398. 10.1080/00222930802586016

[B88] Rivière PG (1966) Some Ethnographic Problems of Southern Guyana. Folk 8–9: 301.

[B89] Rossi Belgrano A, Rossi Belgrano M (2018) Juan Brèthes (Frère Judulien Marie): primer entomólogo del Museo Nacional: colaborador de Florentino Ameghino: vida, obra y familia del ilustre naturalista. Alejandro Alberto Rossi, Buenos Aires, Argentina, 158 pp.

[B90] Sampaio A, da Fonseca CRV (2023) Catalog of the Chrysomelinae (Coleoptera: Chrysomelidae) deposited in the entomological collections of the Instituto Nacional de Pesquisas da Amazônia (INPA) and the Universidade Federal do Amazonas (UFAM), Manaus, Brazil, with an illustrated key for the genera occurring in Brazil (except *Aeneolucentia*, *Jermaniella*, and *Pandona*). Zootaxa 5351: 37–71. 10.11646/zootaxa.5351.1.2 38221498

[B91] Sampaio A, Viana JH, da Fonseca CRV (2024) Catalog of the Chrysomelinae (Coleoptera: Chrysomelidae) deposited in the entomological collections of the Museu Paraense Emílio Goeldi (MPEG) and the Universidade do Estado do Pará (UEPA), Belém, Brazil. Zootaxa 5447: 301–354. 10.11646/zootaxa.5447.3.1 39645827

[B92] Scudder SH (1882) Nomenclator Zoologicus. An alphabetical list of all generic names that have been employed by naturalists for recent and fossil animals from the earliest times to the close of the year 1879. II. Universal index. Vol. 19, Government Printing Office, Washington, D.C., U.S.A., 340 pp. 10.5962/bhl.title.1143

[B93] Seeno TN, Wilcox JA (1982) Leaf beetle genera (Coleoptera: Chrysomelidae). Entomography Publications, Sacramento, CA, USA, 222 pp.

[B94] Sherborn CD (1927) Sect. 2, Pt. 14 (1801-1850), 3393–3746, laminella – Lyzzia. In: Index Animalium. British Museum, London, UK, 354.

[B95] Skelley PE (2020) Nomenclatural notes for the Erotylinae (Coleoptera: Erotylidae). Insecta Mundi 0767: 1–35. 10.5281/zenodo.5353590 34186634

[B96] Société Cuvierienne (1839) Nouveaux membres admis dans la Société Cuvierienne. Revue Zoologique: 64.

[B97] Stål C (1858) Till kännedomen om Amerikas Chrysomeliner. Ofversigt af Kongliga Svenska Vetenskaps-Akademiens Förhandlingar 15: 469–478.

[B98] Stål C (1860) Till kännedomen om chrysomelidae. Ofversigt af Kongliga Svenska Vetenskaps-Akademiens Förhandlingar 17: 455–470.

[B99] Stål C (1865) Monographie des chrysomélides de l'Amérique. III. Nova acta Regiae Societatis Scientiarum Upsaliensis 3(5): 175–365.

[B100] Stephens J (1835) *Phaedon*. In: Smedley E, Rose HJ, Rose HJ (Eds) Encyclopaedia metropolitana; or, universal dictionary of knowledge, on an original plan: comprising the twofold advantage of a philosophical and an alphabetical arrangement, with appropriate engravings Volume XXIII. B. Fellowes, London, United Kingdom, 296–296.

[B101] Strand E (1918) Übersicht der in Gistel’s “Achthundert und zwanzig neue oder unbeschriebene wirbellose Thiere” (1857) behandelten Insekten. Archiv für Naturgeschichte Berlin 82: 75–97.

[B102] Sun X, Marshall SA (2003) Systematics of *Phasia* Latreille (Diptera: Tachinidae). Zootaxa 276: 1–320. 10.11646/zootaxa.276.1.1

[B103] Vincent C, Garambois S, Thibert E, Lefèbvre E, Le Meur Е, Six D (2010) Origin of the outburst flood from Glacier de Tête Rousse in 1892 (Mont Blanc area, France). Journal of Glaciology 56: 688–698. 10.3189/002214310793146188

[B104] Viswajyothi K, Clark SM (2022) New World genera of Galerucinae Latreille, 1802 (tribes Galerucini Latreille, 1802, Metacyclini Chapuis, 1875, and Luperini Gistel, 1848): an annotated list and identification key (Coleoptera: Chrysomelidae). European Journal of Taxonomy 842: 1–102. 10.5852/ejt.2022.842.1945

[B105] Weise J (1915) Übersicht der Chrysomelini. Deutsche entomologische Zeitschrift 1915: 434–436. 10.1002/mmnd.191519150410

[B106] Weise J (1916) Chrysomelidae: 12. Chrysomelinae. In: Schenkling S (Ed) Coleopterum Catalogus. Berlin, Germany, 1–255.

[B107] Welter-Schultes FW (2013) Guidelines for the capture and management of digital zoological names information. Version 1.1 released on March 2013. Global Biodiversity Information Facility, Copenhagen, 126 pp.

[B108] Zaragoza-Caballero S, Zurita-García ML, Ramírez-Ponce A (2023) The on–off pattern in the evolution of the presence of bioluminescence in a derived lineage from fireflies of Mexico (Coleoptera, Lampyridae). Zoologischer Anzeiger 302: 266–283. 10.1016/j.jcz.2022.12.009

